# Comparison of Two Stationary Phases for the Determination of Phytosterols and Tocopherols in Mango and Its By-Products by GC-QTOF-MS

**DOI:** 10.3390/ijms18071594

**Published:** 2017-07-22

**Authors:** Ana López-Cobo, Beatriz Martín-García, Antonio Segura-Carretero, Alberto Fernández-Gutiérrez, Ana María Gómez-Caravaca

**Affiliations:** 1 Department of Analytical Chemistry, Faculty of Sciences, University of Granada, Avd. Fuentenueva s/n, 18071 Granada, Spain; analc@ugr.es (A.L.-C.); bearu15@correo.ugr.es (B.M.-G.); ansegura@ugr.es (A.S.-C.); albertof@ugr.es (A.F.-G.); 2 Functional Food Research and Development Center, Health Science Technological Park, Avd. del Conocimiento, Bioregion Building, 18100 Granada, Spain

**Keywords:** mango, GC-QTOF-MS, phytosterols, tocopherols, by-product, unsaponifiable

## Abstract

Two different gas chromatography coupled to quadrupole-time of flight mass spectrometry (GC-QTOF-MS) methodologies were carried out for the analysis of phytosterols and tocopherols in the flesh of three mango cultivars and their by-products (pulp, peel, and seed). To that end, a non-polar column ((5%-phenyl)-methylpolysiloxane (HP-5ms)) and a mid-polar column (crossbond trifluoropropylmethyl polysiloxane (RTX-200MS)) were used. The analysis time for RTX-200MS was much lower than the one obtained with HP-5ms. Furthermore, the optimized method for the RTX-200MS column had a higher sensibility and precision of peak area than the HP-5ms methodology. However, RTX-200MS produced an overlapping between β-sitosterol and Δ^5^-avenasterol. Four phytosterols and two tocopherols were identified in mango samples. As far as we are concerned, this is the first time that phytosterols have been studied in mango peel and that Δ^5^-avenasterol has been reported in mango pulp. α- and γ-tocopherol were determined in peel, and α-tocopherol was the major tocopherol in this fraction (up to 81.2%); however, only α-tocopherol was determined in the pulp and seed. The peel was the fraction with the highest total concentration of phytosterols followed by seed and pulp, and “Sensación” was the cultivar with the highest concentration of total phytosterols in most cases. There were no significant differences between quantification of tocopherols with both columns. However, in most cases, quantification of phytosterols was higher with RTX-200MS than with HP-5ms column.

## 1. Introduction

Mango (*Mangifera indica* L.), a member of Anacardiaceae family in the order of Sapindales is native to Southeast of Asia and is widely cultivated at both tropical and subtropical latitudes [1]. There are more than 70 genera, and 1000 varieties, and it is considered one of the most important tropical fruits [2]. Presently, mango is cultivated on an area of approximately 3.7 million ha worldwide. Besides, mango fruit is in the second position as a tropical crop, only behind bananas in terms of production and acreage used [3].

Mango fruits provide energy, dietary fibre, carbohydrates, proteins, fats, and phenolic compounds, which are vital to normal human growth, development, and health. Moreover, it has been well documented that mango fruits are an important source of micronutrients, vitamins such as tocopherols, and other phytochemicals such as phytosterols [4,5,6]. Tocopherols play a role as an in vivo antioxidant since they mainly protect polyunsaturated fatty acids in biological membranes and lipoproteins against oxidative damage. Likewise, phytosterols are structural components of the biological membranes which regulate the fluidity and permeability as well as being intermediaries in the biosynthesis of cellulose and secondary products from plants such as alkaloids [7,8,9]. It is well known that phytosterols produce a wide spectrum of biological activities in human. Particularly, phytosterols are considered an efficacious cholesterol-lowering agent [10,11]. In addition to the free form, phytosterols occur as four types of conjugates in which the 3β-OH group is esterified to a fatty acid or a hydroxycinnamic acid, or glycosylated with a hexose (usually glucose) or a 6-fatty-acyl hexose [12].

Mango puree, slices in syrup, nectar, leather, pickles, canned slices, and chutney are the main industrial products obtained from mango fruits. The main by-products produced in the processing of mango fruits are peels and seeds. Peels represent between 7% and 24% of the total weight of mango fruit [13,14] and depending on the cultivar, kernel represents between 45% and 85% of the seed and approximately 20% of the whole fruit [15,16,17]. These by-products have been recognized as natural sources of bioactive phenolic compounds and their use in a commercial purpose could be an important and sustainable opportunity for reducing pollution from bio-wastes [18].

In the past decade, fats from mango, in particular mango seeds, have been extracted, fractionated, and evaluated. In fact, the interest in mango fat has widely increased due to its unique physicochemical characteristics, which are similar to those of cocoa butter [3,17,19,20]. However, there is no information about the distribution of phytosterols and tocopherols in mango and its by-products.

Phytosterols and tocopherols have previously been studied in mango seeds [5,21,22] and tocopherols have also been studied in mango pulp and peel [23,24]. However, as far as we are concerned, there is only one previous work of phytosterols in mango pulp [6].

Thus, the aim of this study has been the optimization and comparison of two different GC-QTOF-MS methodologies for the analysis of phytosterols and tocopherols in pulp, peel, and seed of three different mango cultivars: “Keitt”, “Osteen”, and “Sensación”. To our knowledge, this is the first time that phytosterols have been studied in mango peel.

## 2. Results and Discussion

### 2.1. Optimization of GC-QTOF-MS Methods

The gas chromatography (GC) methods were optimized using a real sample extract of mango peel in full scan mode.

Temperature of the injection port was checked to establish the considered optimal one. It was ranged between 200 and 300 °C and 280 °C was selected as optimal for both columns. Furthermore, the gradient of temperatures of the oven and the gas flow were tested to choose the best compromise between resolution and analysis time. The initial column temperature was checked for both columns (150–250 °C) and 210 °C was selected for RTX-200MS, whereas 250 °C was chosen for HP-5ms. The higher polarity of RTX-200MS column provided a lower retention of compounds in the stationary phase and a lower resolution among them. Thus, a lower initial temperature was needed to obtain a good separation. After that, an increase of temperature was required until an isothermal step was reached: 260 °C for RTX-200MS and 270 °C for HP-5ms. The elution of the compounds of interest was produced in this step; the analysis time for RTX-200MS was much lower than the one obtained with HP-5ms; however, a proper resolution between peaks was achieved with HP-5ms whereas RTX-200MS produced an overlapping between β-sitosterol and Δ^5^-avenasterol. An isothermal step below 260 °C produced an excessive retention of the peaks, increasing their width and the resolution was not significantly improved. Then, the final column temperature was optimized and 310 °C was selected for both columns.

The parameters related to the mass detection method were optimized, taking into account the area of the mass spectrometry (MS) signal of the standards compounds available (α-tocopherol, β-sitosterol, stigmasterol, campesterol and dihydrocholesterol). These parameters were chosen to get a compromise solution to obtain the maximum signal for most of the peaks detected in the samples under study.

### 2.2. Methods Validation

Four calibration curves were prepared using tocopherol, campesterol, stigmasterol, and β-sitosterol standards. Table 1 summarizes the different parameters of each standard compound for both columns. All calibration curves showed good linearity between different concentrations depending on the analytes in question. The calibration plots reveal good correlation between peak areas and analyte concentrations, and the regression coefficients were always higher than 0.99.

Limit of detection (LOD) and limit of quantitation (LOQ) were calculated according to S/N ratio. LOD was found to be within the range 0.883–1.304 μg/mL whereas LOQ was within 2.942–4.342 μg/mL for HP-5ms column, whilst for RTX-200MS column LOD was within 0.578–0.852 μg/mL and LOQ was within 1.925–2.841 μg/mL. As it can be seen, LOD and LOQ of the method optimized using the RTX-200MS column were lower than those obtained for HP-5ms column. Therefore, it can be said that the optimized method for the analysis of tocopherols and phytosterols using RTX-200MS column has a higher sensibility than the HP-5ms methodology.

Method accuracy was determined at three concentration levels: low (LOD), medium (50 μg/mL), and high (100 μg/mL) (Table 1). The recoveries were very close to 100% (96.1% to 100.4%) for both columns used.

Intraday and interday repeatability were developed besides the relative standard deviations (% RSD) (Table 2). Intra- and interday precision of analysis time were very similar for both columns, whereas the intra- and interday precision of peak area were lower for HP-5ms compared to RTX-200MS.

### 2.3. Identification of Phytosterols and Tocopherols of Mango Fractions

**Figure 1:**
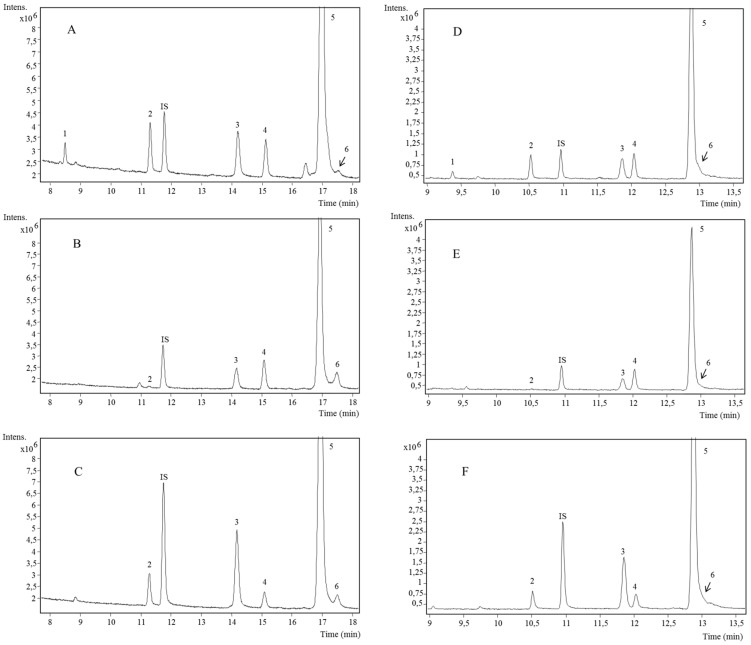
Total ion chromatograms of “Keitt” cultivar obtained using HP-5ms column (**A**) peel, (**B**) seed, (**C**) pulp and using RTX-200MS (**D**) peel, (**E**) seed, (**F**) pulp. See Table 3 for identification numbers. IS (internal standard).

Both optimized methodologies were applied to mango samples and peak identification was done (Table 3 and Figure 1).

Six compounds: two tocopherols and four phytosterols were detected in mango samples. The identification of every compound was corroborated with its standard as exception of γ-tocopherol and Δ^5^-avenasterol. γ and α-tocopherols eluted in the first place. γ-tocopherol (peak 1) at *m*/*z* 488, presented a fragment at *m*/*z* 223 [M-255] due to the loss of the side chain (C_16_H_33_) and another fragment at *m*/*z* 263 [M-(225+40)], because of the cleavage of the side chain accomplished by the breakdown of chroman structure with hydrogen rearrangement and loss of a methyl acetylene fragment [25]. This compound was only detected in mango peel.

γ- and β-tocopherol are isomers; therefore the obtained ions by gas chromatography–mass spectrometry (GC-MS) are the same for both of tocopherol forms [26]. However, in previous studies, the presence of γ-tocopherol and lack of β-tocopherol in mango seed kernel was reported [5]. Indeed, β-tocopherol is the rarest tocopherol homologue present in the plant world with some exceptions, for instance, coffee beans [27] or various types of bran and germ of the wheat [28,29], the presence of γ-tocopherol instead of β-tocopherol can be rather stated in mango.

α-Tocopherol (peak 2), at *m*/*z* 502, showed fragments at *m*/*z* 237 and 277 due to a similar fragmentation pattern previously indicated for γ-tocopherol. α-Tocopherol was detected in mango pulp, peel, and seed. Previous research reports the presence of α-tocopherol in pulp [6].

All of the identified phytosterols were detected in pulp, peel, and seed of mango. The first phytosterol (peak 3) at *m*/*z* 472, was identified as campesterol. It showed three typical fragments: at *m*/*z* 213, due to the loss of the side chain (SC) and the D ring; *m*/*z* 129 and its complement *m*/*z* 343 [M-129]^+^. Other fragments found were *m*/*z* 315 [M-SC-2]^+^, *m*/*z* 255 [M-SC-90]^+^, *m*/*z* 253 [M-SC-90-2]^+^, 457 [M-CH3]^+^, *m*/*z* 382 [M-TMSOH]^+^ (M-trimethylsilyl-OH) and *m*/*z* 367 [M-CH3-TMSOH]^+^. This fragmentation pattern is in agreement with previous literature and also with the spectra of its standard [30,31].

Peak 4 at *m*/*z* 484 and fragments at *m*/*z* 469, 394, 379, 355, 343, 255, 253, 213, and 129, that coincide with the same fragmentation pattern as campesterol, was identified as stigmasterol. This identification was corroborated by the injection of its standard and it is in agreement with Pelillo et al. [30].

β-sitosterol was detected at *m*/*z* 486 (peak 5) and differents fragments at *m*/*z* 471, 396, 381, 343, 357, 255, 253, 213, and 129 were detected according to Pelillo et al. [30]. The identification was also confirmed by its standard.

Campesterol, stigmasterol, and β-sitosterol were previously reported in mango pulp and seed [5,6].

Last phytosterol determined in mango was Δ^5^-avenasterol (peak 6) at *m*/*z* 484 and with the typical fragments at 469, 394, 379, 355, 343, 255, 253, 213, and 129. Besides, it showed an important fragment at *m*/*z* 386 corresponding to [M-98]^+^. This compound was previously reported in seed [5] and, as far as we are concerned, this is the first time that Δ^5^-avenasterol has been identified in mango peel and pulp.

On the one hand, it is interesting to highlight that RTX-200MS column was not able to clearly separate Δ^5^-avenasterol in any fraction of mango. On the other hand, total time of analysis was almost 5 min shorter using RTX-200MS than HP-5ms.

To our knowledge, this is the first time phytosterols have been analyzed in mango peel; therefore, this is the first time that these compounds have been identified and quantified in mango peel. Furthermore, as far as we are concerned, Δ^5^-avenasterol has been reported in mango pulp for the first time in this work.

### 2.4. Quantification of Phytosterols and Tocopherols of Mango Fractions

The methods performed were used to quantify phytosterols and tocopherols in mango pulp, peel, and seed. The results of the phytosterols and tocopherols quantification in different mango fractions and cultivars are showed in Table 4.

In relation to tocopherols, they were present in peel, pulp, and seed; however, peel exhibited higher total concentration of tocopherols than pulp and seed. The concentration of tocopherols was 74.4–79.8% higher in peel than in pulp for the different cultivars when HP-5ms was used and 75.5–79.4% when RTX-200MS was used, whereas tocopherol content was 94.69–97.99% higher in peel than in seed for the different cultivars when HP-5ms was used and 95.48–98.13%. Regarding cultivars, “Sensación” presented the highest content in total tocopherols in all the fractions studied, followed by “Keitt” and “Osteen”. α and γ-tocopherol were simultaneously determined in peel, whereas α-tocopherol was only identified in pulp and seed. α-tocopherol was the major tocopherol in peel. Its percentage with HP-5ms column was 77.39%, 72.28%, and 75.10% of the total tocopherols, in “Keitt”, “Osteen”, and “Sensación”, respectively, and with RTX-200MS column was 81.19%, 72.75%, and 73.77% of the total tocopherols, in “Keitt”, “Osteen”, and “Sensación”, respectively.

Regarding phytosterols, peel was also the fraction with the highest total concentration of phytosterols followed by seed and pulp. “Sensación” was the cultivar with the highest concentration of total phytosterols in most of the cases; although, the analyses performed with RTX-200MS column in peel fraction showed the highest amount in “Osteen” cultivar. Furthermore, analyses with HP-5ms column in seed showed the highest total phytosterols concentration in “Keitt” cultivar. The most abundant phytosterol in mango was β-sitosterol. In the HP-5ms column in peel it reached 78.27%, 79.77%, and 78.40% of the total phytosterols in “Keitt”, “Osteen”, and “Sensación”, respectively; and with RTX-200MS column it reached 78.45%, 81.62%, and 76.21% of the total phytosterols in “Keitt”, “Osteen”, and “Sensación”, respectively. Its percentages in seed with the HP-5ms column were 72.41%, 77.04%, and 75.05% of the total phytosterols in “Keitt”, “Osteen”, and “Sensación”, respectively; and 75.14%, 78.37%, and 77.56% of the total phytosterols in “Keitt”, “Osteen”, and “Sensación”, respectively, with RTX-200MS column. Regarding pulp, β-sitosterol percentages with the HP-5ms column were 71.07%, 70.29%, and 69.83% of the total phytosterols in “Keitt”, “Osteen”, and “Sensación”, respectively; and with RTX-200MS column they were 71.78%, 73.38%, and 72.23% of the total phytosterols in “Keitt”, “Osteen”, and “Sensación”, respectively.

Concentrations of tocopherols and phytosterols in mango are in the same range of concentrations for results found by Vilela et al. [6] in pulp and by Jin et al. [32] in seed. However, differences observed could mainly be attributed to agronomical and environmental factors. The cultivar can also highly influence the appearance and content of tocopherols in fruit seeds [33,34,35].

It is important to notice that there was no difference between quantification of tocopherols with both columns. However, in most cases, quantification of phytosterols was higher with RTX-200MS column than with HP-5ms column.

Statistical analyses were used to determine the correlation between quantifications carried out with HP-5ms and RTX-200MS columns. The results showed that there was a high correlation between total tocopherol and total phytosterol concentration found with HP-5ms and RTX-200MS columns, respectively (*r* = 0.9931; *p* < 0.01 and *r* = 0.9707; *p* < 0.01, respectively).

## 3. Materials and Methods

### 3.1. Samples

Mangoes were provided by Miguel García Sánchez e Hijos, S.A. (Motril, Spain) from the October 2015 harvest. About 10 kg of mango fruits of the cultivars “Keitt”, “Osteen”, and “Sensación” were manually separated in peel, pulp, and seed. The samples were freeze-dried in a lyophilizer (Advantage Plus EL-85 freeze dryer, SP Scientific, Ipswich, Suffolk, UK) and, then, milled and kept at −18 °C until use.

### 3.2. Chemicals and Reagents

Ethanol, potassium hydroxide, diethyl ether, n-hexane, and isopropanol were purchased from Fisher Scientific (Leicestershire, UK). Double-deionized water with conductivity lower than 18.2 MΩ was obtained with a Milli-Q system from Millipore (Bedford, MA, USA). The following phenolic standards and reagents were supplied by Sigma-Aldrich (St. Louis, MO, USA): α-tocopherol, β-sitosterol, stigmasterol, campesterol, dihydrocholesterol. Pyridine was purchased from VWR Chemicals Prolabo (Fontenay-sous-Bois, France). Trimethylchlorosilane and anhydrous sodium sulphate were supplied by Merk KGaA (64271 Darmstadt, Germany), and hexamethyldisilazane was supplied by Alfa Aesar GmbH & Co KG (Karlsruhe, Germany).

### 3.3. Isolation of Unsaponifiable Fraction by Hot Saponification

Hot saponification was performed according to Caligiani et al. [21] with some modifications. Briefly, 1.0 mL of dihydrocholesterol (*c* = 1.0 mg/mL) was added to 15 g of sample powder and saponification was carried out by boiling and stirring for 1 h with 100 mL of 1.0 N potassium hydroxide in ethanol–water solution (4/1 *v*/*v*). The sample was transferred to a separating funnel and 100 mL of distilled water was added. Then, an extraction was carried out with 50 mL of diethyl ether which was repeated four times. The ether extracts were pooled into a separating funnel and washed four times with 50 mL of distilled water. The organic phase was dried with anhydrous sodium sulphate, filtered, dried in a rotary evaporator, and then the residue was weighed and kept at −18 °C until use.

### 3.4. Determination of Phytosterols and Tocopherols by GC-QTOF-MS

Phytosterols and tocopherols were analyzed in a gas chromatograph coupled to a quadrupole-time of a flight mass spectrometer (GC-QTOF-MS) (Agilent Technologies, Santa Clara, CA, USA) consisting of a gas chromatograph (7890B Agilent Technologies), a QTOF mass spectrometer (7200 Agilent Technologies), and an autosampler (GC Sampler 120 Agilent Technologies).

Two methodologies were developed to separate the phytosterols and tocopherols from the different parts of mango by GC-QTOF-MS. To that end, two GC columns were used: HP-5ms (Agilent Technologies, Santa Clara, CA, USA) and RTX-200MS (Restek Corporation, Bellefonte, PA, USA). HP-5ms is a non-polar column with very low bleed characteristics; this column has been widely used for the determination of phytosterols and tocopherols. RTX-200MS is a mid-polar column that offers exceptional thermal stability, low bleed, and superior inertness, even for active compounds. This column has also been used for the analysis of phytosterols in recent years [36].

Quantification was carried out with both HP-5ms and RTX-200MS columns. Four calibration curves were prepared using the following standards: tocopherol, campesterol, stigmasterol, and β-sitosterol. Dihydrocholesterol was used as internal standard (IS). Calibration curves were built using the standard/IS peak area ratio versus standard concentration. Standards were injected under the optimum conditions of the method found for both columns.

LOD and LOQ were calculated based on S/N (Signal to Noise) ratio. They were established injecting the standard solutions by the injection of the smallest amounts which provide S/N = 3 for LOD and S/N = 10 for LOQ. Method accuracy was determined by the closeness of the test value to the nominal value and was evaluated with separately prepared individual primary stock solutions, mixtures, and working solutions of all standards. It was calculated over the linear dynamic range at three concentration levels: low (0.1 μg/mL), medium (50 μg/mL), and high (100 μg/mL) via three assays per concentration on different days. The analyte concentrations were calculated from calibration curves and accuracy was calculated by the ratio of this calculated concentration versus the theoretical one.

Moreover, intraday and interday repeatability were developed to assess the performances of the method, taking into account the standards at the three levels of concentration. Because of that, one mango extract was injected (*n* = 3) three times a day (intraday precision), and for seven consecutive days (interday precision, *n* = 21). The relative standard deviations (% RSD) of analysis time and peak area were determined for RTX-200MS and HP-5ms columns.

Before the analysis, the samples were silylated according to Sweeley et al. [37]. Briefly, silylation was performed adding 200 μL of a mixture containing pyridine/hexamethyldisilazane/trimethylchlorosilane 5/2/1 *v*/*v*/*v* to 5 mg of unsaponifiable fraction. The sample was agitated and incubated at 40 °C for 20 min. Afterwards, it was dried and reconstituted in 2 mL of n-hexane. Finally, the solution was centrifuged at 3500 rpm and 4 °C for 10 min. Separations were carried out with different methods depending on the capillary column used.

#### 3.4.1. HP-5ms Column

The first column used was an HP-5ms (30 m × 0.25 mm inner diameter, 0.25 μm film thickness) from Agilent Technologies. The chromatographic conditions of the method were as follows: initial temperature 250 °C; a temperature gradient of 10 °C·min*^−^*^1^ until reaching 270 °C and holding that temperature for 25 min; a temperature gradient of 10 °C·min*^−^*^1^ until reaching 310 °C and holding that temperature for 5 min;. The injector temperature was 280 °C and the transfer line temperature was 300 °C. The injection volume was 1 μL in split mode with a 1:10 split ratio. The carrier gas was helium at a flow rate 1.2 mL/min.

#### 3.4.2. RTX-200MS Column

The second column was an RTX-200MS (30 m × 0.25 mm inner diameter, 0.25 μm film thickness) from Restek Corporation. The chromatographic conditions of the method were as follows: initial temperature 210 °C; a temperature gradient of 15 °C·min*^−^*^1^ until reaching 260 °C and holding that temperature for 10 min; a temperature gradient of 15 °C·min*^−^*^1^ until reaching 310 °C and holding that temperature for 2 min. The injector temperature was 280 °C and the transfer line temperature was 300 °C. The injection volume was 1 μL in split mode with 1:50 split ratio. The carrier gas was helium at a flow rate 1.2 mL/min.

Instrumental conditions used for the MS detector were as follows: acquisition mode, total ion current; ion source temperature, 230 °C; detector voltage, 70 eV; scan range, from 50 to 700 *m*/*z*; scan speed, 1250 μ/s; solvent delay time, 6 min. Data were filed and processed by the software, MassHunter Workstation (Agilent Technologies, Santa Clara, CA, USA).

## 4. Conclusions

Two methodologies have been developed for the determination of tocopherols and phytosterols in pulp, peel, and seed of three mango cultivars (“Keitt”, “Osteen”, and “Sensación”) by GC-QTOF-MS using two columns with different stationary phases. The analysis time was shorter using the RTX-200MS column than with the HP-5ms. Moreover, the optimized RTX-200MS method could obtain a higher sensibility and precision of peak area than the HP-5ms methodology. However, the RTX-200MS column gave cause to an overlapping between β-sitosterol and Δ^5^-avenasterol.

Four phytosterols and two tocopherols were identified in the different mango fractions studied and no significant differences were found for the quantification results of tocopherols obtained with the two columns. Nevertheless, in most cases, quantification of phytosterols was higher with the RTX-200MS column than with the HP-5ms column. Indeed, the results showed that there was a high correlation between total tocopherol and total phytosterol concentrations found with the HP-5ms and RTX-200MS columns.

Mango fat has demonstrated similar characteristics to cacao butter; however, there is a lack of information about the distribution of some of the families of mango fat in mango fruit. Thus, this work provides evidence about the distribution of phytosterols and tocopherols in mango pulp, peel, and seed. Besides, this is the first time that phytosterols have been studied in mango peel and that Δ^5^-avenasterol has been reported in mango pulp.

## Figures and Tables

**Table 1:** Analytical parameters of the method proposed.

Analyte	Column	Calibration Range (μg/mL)	Calibration Equations	*R*^2^	LOD (μg/mL)	LOQ (μg/mL)	Accuracy (% RSD)		
							LOD μg/mL	50 μg/mL	100 μg/mL
Campesterol	HP5	LOD-100	y = 175,991x – 692,519	0.9962	0.883	2.942	99.3	97.5	99.7
	RTX	LOD-100	y = 245,482x – 89,617	0.9913	0.578	1.925	99.9	98.8	99.1
Stigmasterol	HP5	LOD-100	y = 172,642x – 699,942	0.9961	0.900	2.999	100.1	99.4	99.6
	RTX	LOD-100	y = 325,223x – 200,715	0.9900	0.589	1.963	99.7	100.2	99.9
β-sitosterol	HP5	LOD-100	y = 119,251x + 444,578	0.9944	1.304	4.342	98.2	96.1	100.4
	RTX	LOD-100	y = 180,878x – 101,182	0.9952	0.852	2.841	99.0	98.3	99.5
α-tocopherol	HP5	LOD-100	y = 124,615x – 807,670	0.9958	1.247	4.155	98.7	99.2	97.6
	RTX	LOD-100	y = 186,088x – 178,465	0.9989	0.816	2.719	97.6	99.3	98.7

**Table 2:** Intraday and interday repeatability (expressed as % RSDs) of the area and retention time of the compounds in the HP-5ms and RTX-200MS columns.

Compound	HP5				RTX			
	Intraday RT	Intraday Peak Area	Interday RT	Interday Peak Area	Intraday RT	Intraday Peak Area	Interday RT	Interday Peak Area
α-tocopherol	0.06–0.10	0.84–1.97	0.17–0.23	2.53–3.14	0.01–0.03	0.99–2.41	0.015–0.023	1.27–2.14
Campesterol	0.04–0.09	0.05–0.08	0.019–0.22	1.35–1.76	0.005–0.02	0.98–1.49	0.009–0.017	1.51–1.99
Stigmasterol	0.02–0.13	0.47–0.68	0.20–022	1.29–1.54	0.02–0.03	0.85–1.73	0.013–0.027	1.67–1.96
β-sitosterol	0.02–0.07	0.52–0.71	0.19–0.23	1.31–1.50	0.01–0.03	0.97–1.4	0.014–0.025	1.87–2.61

RT (retention time).

**Table 3:** Phytosterols and tocopherols identification in mango pulp, peel, and seed by GC-QTOF-MS.

Peak	Proposed Compound	Retention Time (min)		*m*/*z* Experimental (M^+^)		*m*/*z* Calculated (M^+^)	Fragments	Molecular Formula	Pulp	Peel	Seed
		HP5	RTX	HP5	RTX						
1	γ-tocopherol	8.47	9.368	488.4010	488.4037	488.4050	263.1471/223.1152	C_31_H5_6_O_2_Si	-	√	-
2	α-tocopherol	11.282	10.518	502.4170	502.4170	502.4206	277.1631/237.1314	C_32_H_58_O_2_Si	√	√	√
3	Campesterol	14.201	11.860	472.4074	472.4095	472.4100	457.3884/382.3610/367.3373/343.3367/253.0179/213.1652/129.0732	C_31_H5_6_OSi	√	√	√
4	Stigmasterol	15.110	12.040	484.4070	484.4073	484.4100	469.3874/394.3609/379.3337/355.3370/343.3362/255.2116/253.0182/213.1650/129.0731	C_32_H5_6_OSi	√	√	√
5	β-sitosterol	17.029	12.882	486.4220	486.4243	486.4257	471.4044/396.3770/381.3535/343.3363/357.3531/255.2121/253.0179/213.1653/129.0745	C_32_H_58_OSi	√	√	√
6	Δ^5^-avenasterol	17.523	12.999	484.4070	484.4075	484.4100	469.3871/394.3605/386.2977/379.3334/355.3368/343.3365/255.2107/253.0182/213.0180/129.0737	C_32_H5_6_OSi	√	√	√

√ Identified in the mango fraction.

**Table 4:** Phytosterols and tocopherols quantification in mango pulp, peel and seed of “Keitt”, “Osteen”, and “Sensación” cultivars measured with HP5 and RTX columns by GC-QTOF-MS expressed as μg/g dry matter.

Compounds		Peel			Pulp			Seed		
		Keitt	Osteen	Sensación	Keitt	Osteen	Sensación	Keitt	Osteen	Sensación
γ-tocopherol	HP5	6.13 ± 0.41b	7.09 ± 0.17c	14.41 ± 0.48d	n.d.	n.d.	n.d.	n.d.	n.d.	n.d.
	RTX	5.19 ± 0.20b	6.22 ± 0.07c	17.73 ± 0.60d	n.d.	n.d.	n.d.	n.d.	n.d.	n.d.
α-tocopherol	HP5	20.99 ± 0.74f	18.47 ± 0.39e	43.45 ± 1.38g	6.72 ± 0.17c	5.16 ± 0.14b	14.81 ± 0.26d	0.98 ± 0.04a	0.87 ± 0.09a	1.02 ± 0.08a
	RTX	22.39 ± 1.06e	16.61 ± 0.31b	49.86 ± 1.20f	6.62 ± 0.69d	4.71 ± 0.17c	16.56 ± 0.40b	0.78 ± 0.08a	0.75 ± 0.08a	0.93 ± 0.09a
TOTAL TOCOPHEROLS	HP5	27.12 ± 1.15c	25.56 ± 0.56c	57.86 ± 1.86e	6.72 ± 0.17b	5.16 ± 0.14b	14.81 ± 0.26d	0.98 ± 0.04a	0.87 ± 0.09a	1.02 ± 0.08a
	RTX	27.58 ± 1.26f	22.83 ± 0.38e	67.59 ± 1.80g	6.62 ± 0.69c	4.71 ± 0.17b	16.56 ± 0.40d	0.78 ± 0.08a	0.75 ± 0.08a	0.93 ± 0.09a
Campesterol	HP5	62.89 ± 0.76c	58.80 ± 1.43b	57.16 ± 1.57b	51.84 ± 0.43f	45.06 ± 1.11e	62.62 ± 0.73c	39.09 ± 1.36d	25.53 ± 0.32a	27.79 ± 1.56a
	RTX	58.25 ± 2.38c	54.01 ± 2.51ac	53.06 ± 2.30a	52.82 ± 1.71a	45.10 ± 0.78e	63.04 ± 1.78f	39.23 ± 1.44d	25.50 ± 0.89b	28.97 ± 2.18b
Stigmasterol	HP5	47.84 ± 4.54c	42.40 ± 0.75b	61.62 ± 0.94f	8.48 ± 0.42a	7.21 ± 0.40a	14.23 ± 0.41f	47.73 ± 1.87c	42.02 ± 0.55b	35.37 ± 1.31e
	RTX	57.38 ± 1.96b	42.54 ± 1.96f	86.09 ± 3.31g	14.61 ± 1.09d	8.15 ± 0.32c	20.77 ± 1.51e	57.33 ± 2.14b	51.58 ± 1.04a	49.82 ± 3.67a
β-sitosterol	HP5	442.92 ± 18.11e	473.89 ± 9.07d	472.89 ± 9.07d	180.49 ± 3.59a	172.24 ± 1.15a	239.88 ± 1.83b	299.56 ± 8.47c	244.61 ± 3.21b	293.19 ± 5.83c
	RTX	504.65 ± 12.77c	565.28 ± 15.34f	497.59 ± 11.01c	217.66 ± 3.02a	218.29 ± 2.58a	290.25 ± 11.43b	382.73 ± 6.23d	307.68 ± 6.00b	421.81 ± 11.03e
Δ^5^-avenasterol	HP5	12.23 ± 0.91a	18.91 ± 0.44b	11.55 ± 0.93a	13.15 ± 0.36a	20.52 ± 1.44b	26.79 ± 0.82c	27.31 ± 0.68c	5.36 ± 0.09d	34.30 ± 2.39e
	RTX	22.98 ± 1.76ad	30.73 ± 2.38ab	16.16 ± 0.96c	18.13 ± 1.28cd	25.94 ± 2.82ab	27.76 ± 2.29ab	30.08 ± 2.57b	7.87 ± 0.78e	43.27 ± 4.55f
Total Phytosterols	HP5	565.88 ± 24.32e	594.00 ± 11.69f	603.41 ± 12.37g	253.96 ± 4.80a	245.03 ± 4.10a	343.52 ± 3.79d	413.69 ± 12.38b	317.52 ± 4.17c	390.65 ± 11.09b
	RTX	643.26 ± 18.87f	692.56 ± 22.19c	652.90 ± 21.98c	311.44 ± 7.10a	297.48 ± 6.50a	401.82 ± 17.01b	509.37 ± 12.38d	392.63 ± 8.71b	543.87 ± 21.43e

Different letters in the same raw indicate significant differences (*p* < 0.05). Three replicates of each sample were analysed.
